# Mitochondria Targeted Viral Replication and Survival Strategies—Prospective on SARS-CoV-2

**DOI:** 10.3389/fphar.2020.578599

**Published:** 2020-08-28

**Authors:** Priya Gatti, Hema Saranya Ilamathi, Kiran Todkar, Marc Germain

**Affiliations:** ^1^Groupe de Recherche en Signalisation Cellulaire and Département de Biologie, Médicale, Université du Québec à Trois-Rivières, Trois-Rivières, QC, Canada; ^2^Centre d’Excellence en Recherche sur les Maladies Orphelines - Fondation Courtois, Université du Québec à Trois-Rivières, Trois-Rivières, QC, Canada

**Keywords:** SARS-CoV-2, mitochondria, mitochondrial dynamics, metabolism, immune response, RNA viruses

## Abstract

SARS-CoV-2 is a positive sense RNA coronavirus that constitutes a new threat for the global community and economy. While vaccines against SARS-CoV-2 are being developed, the mechanisms through which this virus takes control of an infected cell to replicate remains poorly understood. Upon infection, viruses completely rely on host cell molecular machinery to survive and replicate. To escape from the immune response and proliferate, viruses strategically modulate cellular metabolism and alter subcellular organelle architecture and functions. One way they do this is by modulating the structure and function of mitochondria, a critical cellular metabolic hub but also a key platform for the regulation of cellular immunity. This versatile nature of mitochondria defends host cells from viruses through several mechanisms including cellular apoptosis, ROS signaling, MAVS activation and mitochondrial DNA-dependent immune activation. These events are regulated by mitochondrial dynamics, a process by which mitochondria alter their structure (including their length and connectivity) in response to stress or other cues. It is therefore not surprising that viruses, including coronaviruses hijack these processes for their survival. In this review, we highlight how positive sense RNA viruses modulate mitochondrial dynamics and metabolism to evade mitochondrial mediated immune response in order to proliferate.

## Introduction

The COVID-19 pandemic outbreak caused by the novel coronavirus SARS-CoV-2 has infected around 18.5^1^ million people globally as of August 2020, causing severe loss of human life and economic turmoil (Rothan and Byrareddy, 2020). Extensive research is ongoing to develop a vaccine and effective treatment strategies against SARS-CoV-2 infection. However, the mechanisms by which the virus replicates and alters immune responses are not fully established, posing challenges for treatment development.

In eukaryotic cells, mitochondria are the main source of cellular energy, but also control key cellular processes associated with metabolism and immune responses. For example, mitochondria controls cell cycle (Antico Arciuch et al., 2012), cellular differentiation (Papa et al., 2019), signal transduction (Bohovych and Khalimonchuk, 2016), cell metabolism (Spinelli and Haigis, 2018), and apoptosis (Wang and Youle, 2009). In addition, mitochondria serve as an anti-viral signaling platform by activating immune responses through MAVS (mitochondrial antiviral signaling) (Seth et al., 2005; Jacobs and Coyne, 2013; Refolo et al., 2020) and initiating cell death (Lei et al., 2009). It is therefore not surprising that viruses target mitochondria to promote viral replication (Chatel-Chaix et al., 2016; Barbier et al., 2017). While limited information is available concerning the intracellular targets used by SARS-CoV-2 to replicate, evidence from related positive-sense RNA [(+)ssRNA] viruses suggests that mitochondria is a major target of this virus. In this review, we summarize how (+)ssRNA viruses manipulate mitochondrial functions and host immunity to stimulate viral replication, and provide possible mechanisms through which SARS-CoV-2 achieves this.

## SARS-CoV-2 Structure and Replication Cycle

(+)ssRNA viruses dominantly infect eukaryotes, causing human diseases such as Hepatitis C (Hepatitis C Virus, HCV), Dengue (Dengue Virus, DENV), and COVID-19 (SARS-CoV-2) (Koonin et al., 2015). Although (+)ssRNA viruses have their own genomic material, it encodes only a limited set of proteins required for viral replication and assembly of viral particles, making them dependent on the host cellular machinery. For example, (+)ssRNA viruses require the host translation machinery to synthesize the proteins necessary for their replication. These proteins are then assembled in a membrane-associated replication complex that replicates viral RNA (Zhang et al., 2019). Most (+)ssRNA virus replication complexes are associated with the endoplasmic reticulum (ER), although this can also occur on the mitochondrial outer membrane (OMM) in Flock house virus (Miller and Ahlquist, 2002; Virgin et al., 2007). Finally, the newly synthesized genome is packed and transported to Golgi vesicles which fuse with the plasma membrane to release new virions (Stertz et al., 2007).

As other (+)ssRNA virus, the SARS-CoV-2 genome codes for the structural proteins that make up the virion (spike (S), envelope (E), membrane glycoprotein (M), nucleocapsid (N)), as well as non-structural proteins and accessory proteins necessary for viral replication (Chan et al., 2020; Gordon et al., 2020). These proteins are multifunctional and serve for viral replication and to manipulate host functions (**Table 1**) (Gordon et al., 2020; Lu et al., 2020).

**Table 1:** Viral proteins involved in modulating host mitochondrial dynamics and function.

Protein Type & Function	Proteins, Virus	Affected proteins	Viral infection/overexpression of viral proteins	Consequences on Cell Physiology	Reference
**Structural Proteins** Essential for viral infection (**S**pike), attachment (**E**nvelope) and assembly of viral components and virion budding (**N**ucleocapsid **M**embrane & **C**ore)	**M Protein** SARS CoV-2 SARS-CoV		Protein over-expression	Mitochondrial metabolism (predicted) Induces apoptosis	(Gordon et al., 2020) (Chan et al., 2007)
	**N Protein** SARS-CoV	Activation of caspase 3	Protein over-expression	Mitochondria mediated apoptosis caused due to increase in ROS and Cytochrome C release	(Zhang et al., 2009) (Padhan et al., 2008)
	**N/E/** **C protein** HCV	Upregulation of Drp1, Parkin activation	Viral infection	Enhances mitochondrial fission, inhibit apoptosis, facilitates persistent infection, promotes mitophagy	(Kim et al., 2014)
**Non-Structural Proteins** Viral RNA replication	**NSP3** HCV	Upregulation of Drp1, targets MAVS in MAMs	Viral infection	Enhances mitochondrial fission, inhibit innate immune responses, and prevents pro-apoptotic effects of NS4a	(Horner et al., 2011)
	**NSP4** SARS-CoV-2	TIM complex	Protein over-expression	Affects protein import machinery or CM formation (predicted)	(Gordon et al., 2020)
	**NSP4a** HCV	Upregulation of Drp1, target MAVS in MAMs	Viral infection	Enhances mitochondrial fission, inhibits innate immune responses	(Horner et al., 2011)
	**NSP4b** DENV/ZIKA DENV HCV	Downregulation Drp1	Viral infection	Enhances mitochondrial fusion, facilitates replication, increases OXPHOS, triggers mitophagy	(Barbier et al., 2017), (Chatel-Chaix et al., 2016)
		MAMs proteins- MFN2, FACL4	Viral infection	Affects metabolism and induces MAVS	(Barbier et al., 2017),
		MAMs proteins	Both	downregulates OXPHOS & metabolism	(Ramage et al., 2015)
	**NSP5** JEV	Mitochondrial trifunctional protein	Both	Impairs β-Oxidation	(Khromykh et al., 2015)
	**NSP5a** HCV	Activation of NF-κB and STAT-3	Protein over-expression	upregulates mitochondrial calcium (Ca2+), increases cellular ROS and expression of survival genes	(Gong et al., 2001)
	**NSP8** SARS-CoV-2		Protein over-expression	Interact with mitochondrial ribosome (predicted)	(Gordon et al., 2020)
	**NSP13** SARS-CoV- 2	TBK1	Protein over-expression	Regulates MAVS signaling (predicted)	(Gordon et al., 2020)
**Accessory proteins** Enhance selective mode of infection	**ORF 3** HEV	Oligomerization of VDAC1	Protein over-expression	Preserves mitochondrial membrane potential and prevents apoptosis	(Moin et al., 2007)
	**ORF3a** SARS-CoV	BAX oligomerization	Protein over-expression	Promote apoptosis	(Padhan et al., 2008)
	**ORF3b** SARS-CoV	Blocks Type 1 IFN expression	Protein over-expression	inhibit MAVS signaling	(Freundt et al., 2009)
	**ORF9b** SARS-CoV SARS-CoV-2	Drp1, LC3, and MAVS	Protein over-expression	Proteasomal degradation of Drp1 enhances mitochondrial fusion, triggers mitophagy, blocks innate immunity	(Shi et al., 2014)
		Interacts with TOM70	Protein over-expression	Modulate immune pathway (predicted)	(Gordon et al., 2020)
	**ORF9C** SARS- CoV-2	Interacts with ETC components NLRX1, NDFIP2	Protein over-expression	OXPHOS (predicted) Regulate MAVS and immune response (predicted)	(Gordon et al., 2020)

During the infectious cycle, viruses significantly depends on host cell metabolites (nucleotides, amino acids, fatty acids) to produce mature virions. Further, they hijack host defense mechanisms to successfully replicate and propagate. Generally, viruses manipulate host metabolism by re-orchestrating organelle structure and functions. As mitochondria has multifaceted roles in controlling cellular metabolism and immune responses, it is not surprising that several (+)ssRNA viruses target mitochondria.

## Multifaceted Organelle: Mitochondria

Mitochondria constitute a key metabolic hub within a cell, being required for ATP synthesis and a large number of metabolic reactions (Kluge et al., 2013). To achieve this, mitochondria are structurally and functionally compartmentalized. The OMM controls mitochondria protein import but also several signaling pathways, including for immune activation (Giacomello et al., 2020; Tiku et al., 2020). On the other hand, invaginations of the inner mitochondrial membrane (IMM) called cristae are the site where the electron transport chain (ETC) creates the electrochemical gradient required for the ATP synthase-dependent production of cellular ATP (Gilkerson et al., 2003; Zorova et al., 2018). In addition to their functional roles, the OMM and IMM define two soluble compartments, the intermembrane space (IMS) and the matrix. While the IMS contains proteins involved in cellular apoptosis and ROS metabolism (Herrmann and Riemer, 2010), the matrix contains mitochondrial DNA (mtDNA) and is a crucial metabolic hub (Spinelli and Haigis, 2018; Yan et al., 2019).

In contrast to this static description, mitochondria are highly dynamic organelles. The dynamic reorganization of mitochondrial structure is essential to maintain cellular homeostasis in response to cues such as metabolic changes or infection. Mitochondrial dynamics, which include mitochondrial fusion and fission, are controlled by a number of large GTPases of the Dynamin family. OMM fusion is mediated by Mitofusins (MFN1,2), while IMM fusion requires OPA1 (Song et al., 2009; Del Dotto et al., 2018). In addition, oligomerization of OPA1 regulates cristae structure and ETC assembly (Patten et al., 2014; Cogliati et al., 2016). Mitochondrial fission occurs at ER-mitochondria contact sites where DRP1 is recruited and oligomerizes to cause mitochondrial scission (Bui and Shaw, 2013; Korobova et al., 2013).

As damaged mitochondria promote the production of harmful reactive oxygen species (ROS) and stimulate inflammation, cells have developed specialized quality control mechanisms to eliminate damaged/depolarized mitochondria. These function by either selectively removing damaged mitochondrial proteins using mitochondria derived vesicles or completely eliminating depolarized mitochondria by a selective autophagy mechanism, termed mitophagy, that requires mitochondrial fission to selectively deliver damaged mitochondria to lysosomes and prevent damaging effects on cells (Twig et al., 2008; Kim and Lemasters, 2011; Soubannier et al., 2012; Shirihai et al., 2015; Fritsch et al., 2020).

Several (+)ssRNA viruses alter ER-mitochondria interactions or manipulate mitochondrial dynamics and mitophagy. In this way, they can manipulate host metabolism and immunity to their advantage. (+)ssRNA viruses that target mitochondria include HCV, DENV, Zika (ZIKV), and coronaviruses related to SARS-CoV-2 [SARS-CoV (SARS) and Middle East respiratory syndrome (MERS)-CoV]. In addition, the recently published interactome of SARS-CoV-2 proteins suggests that SARS-CoV-2 also targets mitochondria (Gordon et al., 2020), highlighting an important role for mitochondria in (+)ssRNA virus biology.

## Viral Control of Mitochondrial Dynamics and Metabolism

Several (+)ssRNA viruses target mitochondrial dynamics. For example, HCV causes DRP1 mediated mitochondrial fission (Kim et al., 2014) and HCV core protein physically interacts with Parkin to stimulate PINK1/Parkin mediated mitophagy, preventing immune activation and apoptosis induction (Kim et al., 2013; Kim et al., 2014). However, in most cases including DENV, ZIKV, and SARS-CoV, the infection causes mitochondrial elongation that is associated with the formation of convoluted membranes (CMs), ER derived membranes required for viral replication (Shi et al., 2014; Chatel-Chaix et al., 2016; Barbier et al., 2017). In DENV, mitochondrial elongation is dependent on the viral protein NS4B, a component of the replication complex which selectively inhibits the fission protein DRP1 (Chatel-Chaix et al., 2016; Barbier et al., 2017). Similarly, SARS-CoV ORF-9b induces mitochondrial elongation through proteasomal degradation of DRP1 (Shi et al., 2014). Importantly, these viruses induce mitochondrial elongation to promote viral replication, as well as to inhibit host immune activation (Shi et al., 2014; Chatel-Chaix et al., 2016; Barbier et al., 2017).

The association between mitochondrial elongation and viral replication is possibly the result of alterations in Mitochondria Associated Membranes (MAMs). MAMs are contact sites between the ER and mitochondria that regulate mitochondrial dynamics, serve to transfer a number of molecules between the two organelles and promote the activation of some pattern recognition receptors (PRR) (including MAVS; see below) (Friedman et al., 2011; Vazquez et al., 2015; Wu and Zou, 2019; Abrisch et al., 2020). In fact, DENV infection disrupt MAMs by downregulating the MAM proteins MFN2 and FACL4 (Barbier et al., 2017), while CM formation further reduces ER-mitochondria contact sites (Chatel-Chaix et al., 2016). Similarly, HCV NS4B protein is predicted to regulate MAMs to promote viral replication (Ramage et al., 2015), while a proteomics analysis of MAMs in chronic HCV infection showed a significant downregulation of proteins involved in oxidative phosphorylation and enzymes involved in energy and lipid metabolism (Wang et al., 2015).

MAM disruption can also disturb intracellular processes including autophagy (Gomez-Suaga et al., 2017), a catabolic process required to recycle amino acids during nutrient starvation and degrade damaged organelles (Lin et al., 2014). Interestingly, some viruses alleviate this by promoting mitophagy of dysfunctional mitochondria through Parkin activation (DENV and ZIKV), or interaction with LC3 (SARS-CoV) (Shi et al., 2014; Datan et al., 2016; Liang et al., 2016). In the case of transmissible gastroenteritis virus, this prevents apoptosis activation (Zhu et al., 2016).

Overall, targeting of mitochondria and MAMs by (+)ssRNA viruses promotes viral replication through CM formation, but also stimulates metabolic alterations. For example, HCV upregulates enzymes involved in glutamate catabolism and TCA cycle enzymes during early stages of infection to facilitate a regular supply of TCA cycle intermediates and lipids (Diamond et al., 2010). As mitochondrial fusion promotes mitochondrial activity, increased mitochondrial respiration (DENV) and Complex-I coupled respiration (Sindbis) that maintain total ATP levels during infection could be the consequence of mitochondrial elongation (Saks et al., 2012; Barbier et al., 2017; Yao et al., 2019).

A number of (+)ssRNA viruses require lipid metabolism for viral genome replication and virions assembly (Zhang et al., 2019). In fact, mitochondrial β-oxidation is essential to replenish metabolic intermediates and to produce the energy required for HCV replication (Diamond et al., 2010; Rasmussen et al., 2011). Similarly, DENV replication is highly dependent on β-oxidation of triglycerides present in lipid droplets (Heaton and Randall, 2010), although this was not affected by knocking down the β-oxidation enzyme dodecanoyl CoA delta isomerase in HCV (Rasmussen et al., 2011).

On the other hand, chronic viral infection can significantly impair mitochondrial function. For example, long-term HCV infection or expression of the HCV polyprotein severely impairs mitochondrial respiration through loss of Complex I and V activity, and decreased β-oxidation caused by PPARα downregulation (Dharancy et al., 2005; Ripoli et al., 2010). HCV-induced loss of mitochondrial function also results in HIF-1α stabilization and a shift to glycolysis (Ripoli et al., 2010). Similarly, Sindbis virus impairs both Complex I and II-linked respiration following replication, resulting in cell death (Saks et al., 2012), while the Japanese Encephalitis Virus (JEV) protein NS5 disrupts β-oxidation through its interaction with hydroxyacyl-CoA dehydrogenase, thereby promoting virulence (Khromykh et al., 2015). Interestingly, transcriptome profiling of MERS and SARS-CoV-infected bronchial epithelial cells also suggested a potential downregulation of the Complex I subunit NDUFA10 (Guzzi et al., 2020). Thus, while (+)ssRNA viruses depend on mitochondrial metabolism to generate the ATP and metabolites required for viral replication and virion assembly, this can be reversed at later time points, leading to metabolic dysfunction and cell death.

## Mitochondria-Dependent Strategies of Immune Regulation by Host Cells and Viruses

Upon viral infection, the immune system springs into action. Initially, viral components (capsid, nucleic acids) are detected by intracellular receptors (PRRs) that activate proinflammatory responses (Sparrer and Gack, 2015; Lee et al., 2019; Matz et al., 2019). Mitochondria regulate viral detection and subsequent immune responses through the activation of the MAVS protein (Vazquez et al., 2015), the release of mtDNA (Wu et al., 2019; Riley and Tait, 2020), or through MAM modulation (Reshi et al., 2018). MAVS activation is a key event following infection with RNA viruses. This pathway is activated when viral RNA is recognized by the RIG-I PRR, triggering its recruitment to MAMs where it interacts with MAVS. This leads to the activation of TBK-1 and NF-kB and subsequent expression of type I interferons (IFNs) and proinflammatory cytokines. By damaging mitochondria and causing mtDNA release to the cytosol, RNA viruses can also indirectly activate the dsDNA-stimulated PRR pathway, cGAS-STING (Sun et al., 2017).

Not surprisingly, PRR pathways and especially MAVS are strongly modulated by (+)ssRNA viruses to promote viral persistence and evade immune responses. For example, the NS3/4A protease of HCV selectively cleaves MAM-associated proteins, thereby suppressing antiviral responses (Horner et al., 2011; Horner and Gale, 2013). Similarly, in SARS-CoV, ORF-9b targets mitochondria where it promotes the degradation of MAVS and some of its downstream effectors (TRAF-3 and -6)(Shi et al., 2014), while ORF 3b blocks the expression of type 1 IFNs (Freundt et al., 2009). Intriguingly, some (+)ssRNA viruses also stimulate the expression of Prohibitins (PHB) (Mukherjee et al., 2017; Too et al., 2018), IMM proteins required to maintain the structural integrity of mitochondria, but also for MAVS activation (Signorile et al., 2019; Yoshinaka et al., 2019). While increasing PHB expression might seem counterintuitive when trying to evade host immunity, its main purpose could be instead to stimulate mitochondrial elongation and activity.

In fact, immune repression by (+)ssRNA viruses is associated with changes in mitochondrial dynamics and MAMs. For example, SARS-CoV ORF-9b causes mitochondrial elongation in addition to MAVS degradation (Shi et al., 2014). A functional link between these events is supported by the observation that knockdown of fission (DRP1) inhibits IFN-dependent responses following DENV infection, while promoting fission (MFN2 knockdown) enhances innate immune responses in the context of DENV and HCV infections (Horner et al., 2011; Chatel-Chaix et al., 2016). Thus, during their initial phase of infection, (+)ssRNA viruses selectively target mitochondria to control cellular metabolism to their advantage, but also to short-circuit mitochondria-dependent immune activation.

One last defense mechanism that cells use to combat pathogens is apoptotic cell death. Following an apoptotic signal, the OMM is permeabilized by pro-apoptotic BCL-2 proteins (BAX, BAK), resulting in the release of cytochrome c into the cytosol and caspase activation (Wang and Youle, 2009; Xiong et al., 2014). However, viruses have evolved multiple evasion strategies to prevent apoptosis, including stimulating the expression of survival genes (i.e. anti-apoptotic BCL-2 proteins) or avoiding apoptosis by counteracting the pro-apoptotic effect of viral proteins. For example, the HCV protein NS3 prevents the pro-apoptotic effect of NS4A (Mottola et al., 2002; Nomura-Takigawa et al., 2006). Other anti-apoptotic mechanisms used by (+)ssRNA viruses to prevent apoptosis during early phases of infection include calcium and ROS-dependent activation of STAT-3 and NF-κB survival pathways (HCV NS5A protein) (Gong et al., 2001) and decreased OMM permeabilization through VDAC1 oligomerization and increased interaction with hexokinase I [Hepatitis E virus (HEV) protein orf3] (Moin et al., 2007). Besides, (+)ssRNA viruses activate mitophagy to eliminate damaged mitochondria and thus prevent apoptosis (Kim et al., 2014; Zhu et al., 2016).

While many viruses have developed strategies to block apoptosis in early phases of infection, it can still be activated at later time points as a consequence of accumulated cellular damage caused by viral replication, immune activation or specific viral proteins. For example, SARS-CoV nucleocapsid and ORF3a can induce apoptosis under some circumstances (Padhan et al., 2008; Zhang et al., 2009). However, apoptosis induction does not affect SARS-CoV replication or its dissemination, suggesting that apoptosis is a downstream effect of enhanced cytokine response (Bordi et al., 2005). Thus, (+)ssRNA viruses evade the harmful effects of apoptosis by inhibiting or delaying its activation.

## SARS-CoV-2 Targets Mitochondria?

As a new coronavirus, the mechanisms by which SARS-CoV-2 control its host remain unclear. Furthermore, the severity of illness due to SARS-CoV-2 infection is likely impacted by both direct cytotoxic effects of the virus and the effectiveness of host responses. Interestingly, a recent gene expression profiling of SARS-CoV-2 infection showed an altered autophagy response, activation of innate immunity, increased ROS processes, and downregulation of mitochondrial function (Singh et al., 2020), suggesting that as with other (+)ssRNA viruses, SARS-CoV-2 manipulates immune responses and metabolism to stimulate its replication.

**Figure 1:**
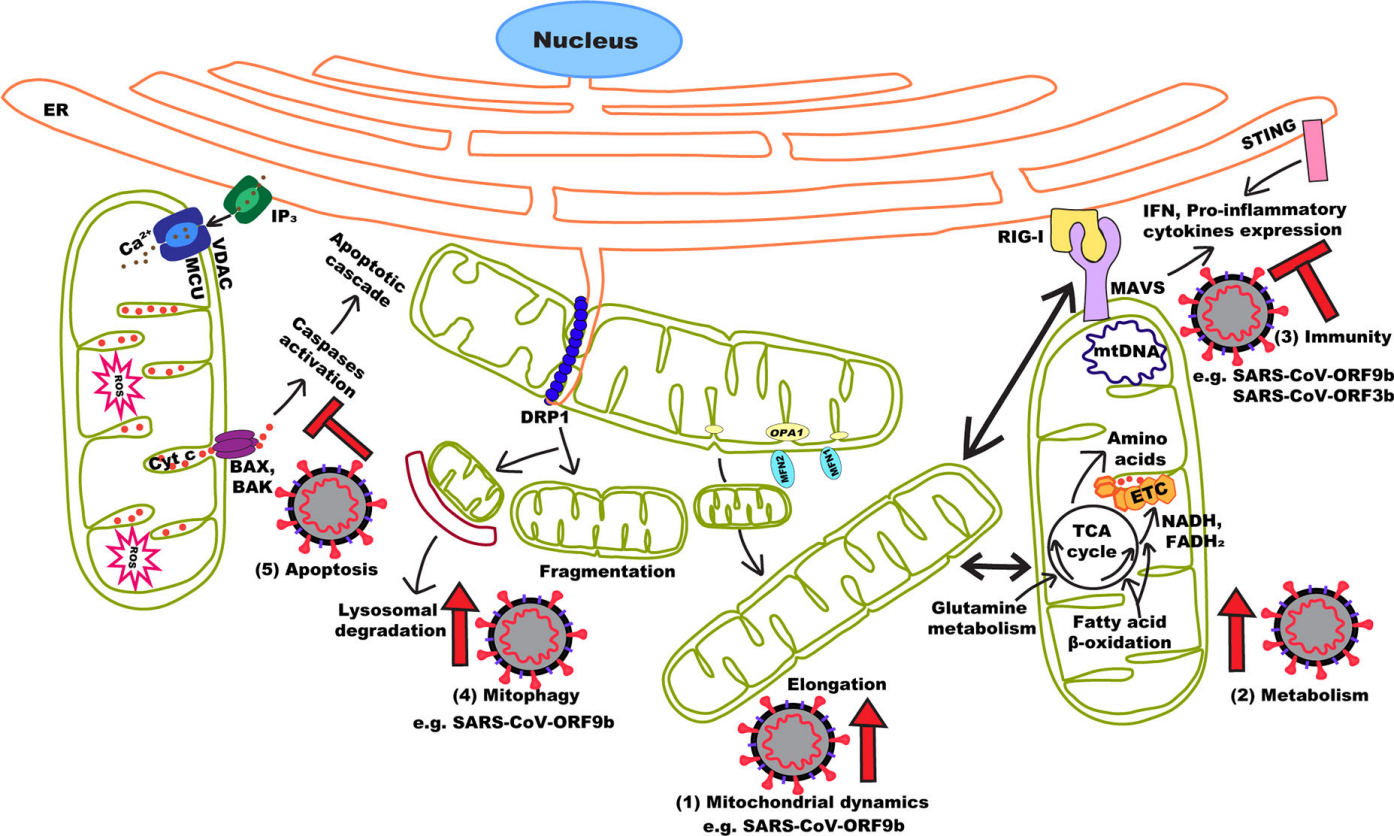
Effects of (+)ssRNA viruses on mitochondrial dynamics and function. By manipulating mitochondrial dynamics (1), (+)ssRNA viruses either directly or indirectly manipulate a number of cellular processes to promote viral replication and immune evasion. These include increasing mitochondrial activity (2), inhibiting MAVS activation (3) and activating mitophagy (4) to prevent apoptosis (5). In the case of SARS-CoV (and probably SARS-CoV-2), the ORF9b protein can perform most of these functions.

In this context, the study of other (+)ssRNA can provide important information on SARS-CoV-2 biology and how it could target mitochondria to control its host (**Figure 1**). Recent data suggest that SARS-CoV-2 uses strategies similar to those of related coronaviruses to manipulate mitochondria and promote its replication. In fact, a number of SARS-CoV-2 proteins potentially interact with mitochondrial proteins. Among these, ORF9c was found to interact with ETC components, suggesting a possible role in regulating OXPHOS (Gordon et al., 2020). SARS-CoV-2 also expresses ORF9b which causes mitochondrial elongation and MAVS degradation in SARS-CoV (Shi et al., 2014). In SARS-CoV-2, ORF9b pulled down the TOM70 subunit of the OMM protein import machinery (Gordon et al., 2020), which also recruits components of the MAVS pathway to the OMM (Liu et al., 2010). SARS-CoV-2 could also regulate MAVS activation through the interaction of ORF9c with negative regulator of MAVS signaling (NLRX1, NDFIP2), or the interaction of Nsp13 with the MAVS effector TBK1 (Gordon et al., 2020).

Intriguingly, SARS-CoV-2 ER-resident protein Nsp4, which is required for CM formation in SARS-CoV (Hagemeijer et al., 2014), potentially interacts with components of the IMM protein import machinery (TIM complex) (Gordon et al., 2020). As mitochondrial dynamics and CM formation are linked in (+)ssRNA viruses like DENV and ZIKV (Chatel-Chaix et al., 2016), Nsp4 could provide a mechanistic link between them. Other potential mitochondria-interacting SARS-CoV-2 proteins include Nsp8 and the M protein. Nsp8 has been reported to interact with components of mitochondrial ribosomes (Gordon et al., 2020) although, since Nsp8 is part of the viral RNA polymerase complex, this could be the consequence of its affinity for RNA (te Velthuis et al., 2012). Similarly, the M protein potentially interacts with a number of proteins involved in mitochondrial metabolism (Gordon et al., 2020). Given that the M protein from SARS-CoV induces apoptosis (Chan et al., 2007), it remains to be determined if these interactions are associated with metabolic regulation or cell death. Altogether, these evidences are consistent with SARS-CoV-2 targeting mitochondria to facilitate its replication.

## Conclusion

Our understanding of SARS-CoV-2 pathogenesis is still incomplete, and the current lack of effective treatment strategies or vaccine makes SARS-CoV-2 infection a major health issue. In this context, analyzing the mechanisms through which other coronaviruses, and more generally (+)ssRNA viruses control their host metabolism and immune activation can provide important information concerning the biology of SARS-CoV-2. Interestingly, the ability of (+)ssRNA viruses to target mitochondria to modulate metabolism and promote the formation of ER-derived replicative structures seems to be conserved in SARS-CoV-2. In addition, these mechanisms likely allow this virus to inhibit MAVS- and STING-dependent immune activation. Targeting these processes could thus provide novel approaches to treat infections caused by SARS-CoV-2 and other (+)ssRNA viruses.

## Author Contributions

All authors contributed to the article and approved the submitted version.

## Funding

This work was supported by grants from the Natural Sciences and Engineering Research Council of Canada and the Fondation UQTR. PG is a recipient of a Queen Elizabeth II Diamond Jubilee scholarship. HSI is a recipient of a Queen Elizabeth II Diamond Jubilee scholarship and a Fonds du Québec- Nature et technologies. KT is a recipient of a Queen Elizabeth II Diamond Jubilee scholarship and a Fonds du Québec-Santé scholarship.

## Conflict of Interest

The authors declare that the research was conducted in the absence of any commercial or financial relationships that could be construed as a potential conflict of interest.

## Footnotes

1 COVID-19 dashboard by the Center for Systems Science and Engineering (CSSE) at Johns Hopkins University. https://arcg.is/0fHmTX [Accessed August 5, 2020]
